# Impact of ataxia-telangiectasia mutated (ATM) loss on radiobiological and immune response to radium-223 in prostate cancer *in vitro* models

**DOI:** 10.1016/j.ctro.2026.101206

**Published:** 2026-05-30

**Authors:** Victoria L. Dunne, Timothy C. Wright, Aislinn Toner, Melissa LaBonte Wilson, Kienan I. Savage, Joe M. O’Sullivan, Kevin M. Prise

**Affiliations:** a Johnston Cancer Research Centre, Queen’s University Belfast, Northern Ireland, UK; b Northern Ireland Cancer Centre, Belfast Health & Social Care Trust, Northern Ireland, UK

**Keywords:** Prostate cancer, Radium-223, X-rays, ATM-depletion, DNA damage, cGAS-STING pathway

## Abstract

Introduction: Personalised medicine approaches are redefining the therapeutic landscape for men with metastatic castration-resistant prostate cancer (mCRPC). While poly(ADP-ribose) polymerase (PARP) inhibitors have demonstrated clinical benefit in patients with BRCA1/2 mutant tumours, the therapeutic efficacy of these inhibitors in Ataxia-telangiectasia mutated (ATM)-mutated prostate cancer patients remains modest, highlighting the need for alternative treatment strategies. This study aimed to elucidate the impact of ATM loss on radiobiological and immune responses to different radiation modalities in prostate cancer models. Methods: Isogenic CRISPR Cas-mediated ATM-deficient and wild-type (WT) cells were treated with X-rays or Radium-223 dichloride (^223^Ra). Cellular radiosensitivity was measured using clonogenic assays and 53BP1 immunofluorescence assessed DNA damage. Cell cycle distribution and apoptosis were analysed by flow cytometry. Innate immune activation was assessed using cGAS immunofluorescence and quantitative PCR of CCL5, CXCL10 and IFIT2. Results: ^223^Ra significantly increased radiosensitivity in comparison to X-rays which was amplified by ATM loss. ^223^Ra exposure in ATM deficient cells induced significantly greater levels of DNA damage as measured by persistent 53BP1 foci at 24h, distinct G2 accumulation and elevated levels of apoptosis in PC-3 and DU145 ATM-deficient cells in comparison to either X-rays or WT counterparts (p < 0.05). Furthermore, ^223^Ra triggered cGAS positive micronuclei and upregulation of STING driven inflammatory genes, particularly in ATM-deficient cells (p < 0.05). Conclusions: These findings demonstrate that ATM-deficiency enhances radiobiological response and amplifies immune activation to ^223^Ra, supporting further pre-clinical evaluation of ATM loss as a determinant of response and therapeutic target to optimise ^223^Ra based strategies for mCRPC.

## Highlights

• ATM-deficiency shows increased radiosensitivity to radium-223 relative to X-rays.
• Radium-223 generates persistent, complex DNA double strand breaks that are inefficiently repaired without ATM.
• ATM-deficiency enhances G2 phase arrest and promotes radiation-induced apoptotsis following radium-223 exposure.
• ATM-deficiency increases cytosolic micronuclei and amplifies cGAS-STING-mediated immune signalling.

## Introduction

Prostate cancer (PCa) is the second most frequently diagnosed cancer and the fifth leading cause of cancer-associated death among men globally, with approximately 375,000 deaths annually [1]. Treatment approaches vary depending on disease stage and aggressiveness, with localised disease often managed effectively with surgery or external beam radiotherapy. However, mCRPC is clinically aggressive, with limited therapeutic options and a median survival of less than 3 years [2].

Radium-223 dichloride (^223^Ra) is an alpha (α)-emitting radiopharmaceutical with a short tissue penetration range (< 100 μM), which selectively targets osteoblastic bone metastases and is approved for the treatment of mCRPC [3]. Upon decay, the release of high linear energy transfer (LET) α-particles induces dense clusters of complex DNA double-strand breaks (DSBs) that are difficult to repair. However, despite the clinical benefit of ^223^Ra, mCRPC remains incurable, highlighting the need for novel therapeutic strategies [4].

Emerging studies have focused on exploiting molecular vulnerabilities in the DNA damage response (DDR) pathway to overcome therapeutic limitations with current treatments. Ataxia-telangiectasia mutated (ATM)-mutated kinase is a member of the PI3K-related serine/threonine family and plays an essential role in guarding genomic integrity through the detection and repair of DSBs, cell cycle checkpoint activation and initiating apoptosis in cases of excessive DNA damage [5], [6]. Loss of, or deficiencies in, ATM occur in 2-5% of patients with advanced PCa and are associated with increased genomic instability, therapeutic resistance and poor clinical outcomes [7], [8], [9], [10], [11]. Importantly, the biological effects of densely ionising radiation in the context of ATM deficiency have been widely investigated, with preclinical studies demonstrating increased sensitivity of ATM-deficient tumour models to high-LET radiation due to impaired repair of complex clustered DNA damage [12], [13]. Consistent with these findings, retrospective studies have demonstrated that patients harbouring deficiencies in homologous recombination repair (HRR)-mediated DNA repair genes, including ATM, derive greater clinical benefit from ^223^Ra compared with patients without DDR alterations, suggesting that DDR-deficient tumours may be particularly susceptible to α-particle-induced clustered DNA damage [14], [15], [16]. Consistent with these findings, we previously demonstrated that pharmacological inhibition of ATM can enhance the therapeutic efficacy of ^223^Ra in PCa *in vitro* models, further supporting ATM as a potential determinant of response to α-particle therapy [17].

Radiotherapy has also been shown to elicit systemic responses in non-irradiated cells that are mediated by inflammatory signalling [18], [19]. Similarly, loss of functional DDR genes in cancer have been associated with activation of innate immune signalling pathways, including the cGAS-STING-mediated pathway [20], [21]. Mechanistically, the accumulation of unrepaired DSBs leads to the formation of cytosolic DNA fragments, which activate the cGAS-STING pathway, driving type 1 interferon signalling and chemokine expression [22]. This response appears increased in cells with deficiencies in key DDR genes, indicating that DDR loss and α-particle induced DNA damage may synergistically potentiate immune activation [20], [21].

The radiobiological and immunological consequences of ^223^Ra treatment in the context of ATM deficiency remains poorly understood. This preclinical study investigated how loss of ATM influences the radiobiological and immune responses to ^223^Ra in PCa, with the aim of informing future combination strategies involving α-particle radiopharmaceuticals and immunotherapy.

## Materials and methods

For Cell Lines, Cell Culture and Irradiation Setups, see Supplementary.

### Clonogenic survival Assay

Colony formation assays were performed according to published methods [23]. Briefly, cells were seeded into six-well plates (Sarstedt AG & Co., Nümbrecht, Germany) at densities tailored for each cell line and absorbed radiation dose. After 24 h, cells were irradiated with X-rays (0-8 Gy) or exposed to ^223^Ra for 24 h to deliver doses of 0-0.5 Gy. Following treatments, cells were incubated for 7-10 days to allow colony formation. Colonies were fixed and stained (2% crystal violet in 80% methanol) with colonies containing > 50 cells scored as surviving cells. Survival fraction was calculated by dividing the colonies formed after irradiation by the number of cells seeded, adjusted for the plating efficiency of untreated cells.

Survival data were fitted to the linear-quadratic model, SF = exp[-(αD + βD^2^)], using non-linear regression, where SF is the survival fraction, D is the dose, and α and β are constants. Sensitisation enhancement ratios (SER) were calculated from the ratios of the mean inactivation dose (MID), determined from the area under the survival curves. RBE was calculated from the ratios of MIDs of radium to X-rays.

Approximate isotoxic doses of different radiation qualities (2 Gy of X-rays or 0.25 Gy of ^223^Ra), defined by clonogenic survival in wild-type cell lines, were employed to assess the effects of ATM loss across the radiobiological and immunological analyses described below.

### Immunofluorescence assays

Cells were seeded at a density of 1 × 10⁶ per well on autoclaved 18 × 18 mm coverslips (Mensel Glaser, Braunschweig, Germany) and allowed to adhere for 24 h before exposure to X-rays (2 Gy) or ^223^Ra (0.25 Gy). Following X-ray irradiation, cells were fixed at 1 h or 24 h post-exposure. For ^223^Ra-treated cells, fixation was performed either 1 h or 24 h following completion of the 24 h exposure period. Cells were fixed with 4% formaldehyde on ice for 30 min, permeabilized with 0.5% Triton X-100, and blocked with 5% FBS/0.1% Triton X-100 in PBS. Immunostaining with anti-53BP1 (NB100-304, Novus Biologicals, Centennial, CO, USA; 1:5000 in blocking buffer) for 1 h at 4 °C was followed by, Alexa Fluor 568 goat anti-rabbit IgG (A21429, Invitrogen, Waltham, MA, USA; 1:2000 in blocking buffer) for 1 h at room temperature in the dark. Nuclei were counterstained and mounted with ProLong Gold antifade reagent containing DAPI (P36930, Invitrogen). Foci were scored using a Zeiss Axiovert 200 M microscope (Carl Zeiss MicroImaging, White Plains, NY, USA) at × 63 magnification and for each treatment condition, 53BPI was analysed in 50 cells. Data are presented as the mean number of foci per cell ± standard error from three independent experiments.

For cGAS staining, cells were blocked with 3% BSA in PBS and immunostained using cGAS (D1D3G) (#15102, Cell Signaling Technology, Danvers, MA, USA; 1:1000 in blocking buffer) at 4 °C overnight. Following washing, Alexa Fluor 488 goat anti-rabbit IgG (A11008, Invitrogen; 1:000 in blocking buffer) was added for 1 h at room temperature in the dark. Slides were then washed, incubated in DAPI for 10 min at room temperature in the dark and mounted with ProLong Gold antifade reagent. Micronuclei were defined as distinct DNA aggregates separate from the primary nucleus in cells [17].

### Flow cytometry

Following irradiations, cells were harvested and fixed in 100% ice-cold ethanol, stored at -20 °C and subsequently resuspended in 360 μL propidium iodide (PI)/RNase A solution. Samples were incubated for 30 min at 37 °C before flow cytometric analysis.

Apoptosis and necrosis were assessed using Annexin V-FITC/PI Apoptosis Detection Kit according to the manufacturer's instructions (Merck Biosciences, Nottingham, UK).

Flow cytometric analyses for both cell cycle distribution and apoptosis were performed using a BD Accuri C6 Plus flow cytometer (BD Biosciences, San Jose, CA, USA). Cell-cycle profiles were quantified using BD Accuri C6 Plus Analysis software (version 1.0.23). Annexin V+/PI- populations were classified as early apoptotic cells, while Annexin V+/PI + populations represent late apoptosis or secondary necrosis.

### Quantitative PCR

Cells were seeded in 6-well plates and allowed to adhere for 24 h before irradiations. At 96 h post-irradiation, cells were lysed in RNA lysis buffer (Qiagen, Hilden, Germany) and total RNA was extracted using the RNeasy Plus Mini Kit (Qiagen) according to manufacturer’s instructions. Complementary DNA (cDNA) was synthesised from total RNA using the First Strand cDNA Synthesis Kit (Roche Diagnostics GmbH, Mannheim, Germany). Quantitative real-time PCR (qPCR) was performed on a LightCycler 480 system (Roche) using SYBR Green Master Mix (Roche) and gene-specific QuantiTect primer assays (Qiagen) for *ACTB* (Hs_ACTB_2_SG; QT01680476), *CCL5* (Hs_CCL5_1_SG; QT00090083), *CD274* (Hs_CD274_1_SG; QT00082775), and *IFIT2* (Hs_IFIT2_2_SG; QT02289294). Relative gene expression was calculated, with *ACTB* serving as the internal reference gene and values were normalised to untreated controls.

### Statistical analysis

All statistical analyses were performed by GraphPad Prism 7.0 (GraphPad, Boston, MA, USA), and the data presented as mean ± standard error. Differences between two groups were compared using unpaired Student’s *t*-tests and considered statistically significant at *p* < 0.05. Statistical significance was shown as *p < 0.05, **p < 0.01, ***p < 0.001, ****p < 0.0001.

## Results

**Fig. 1:**
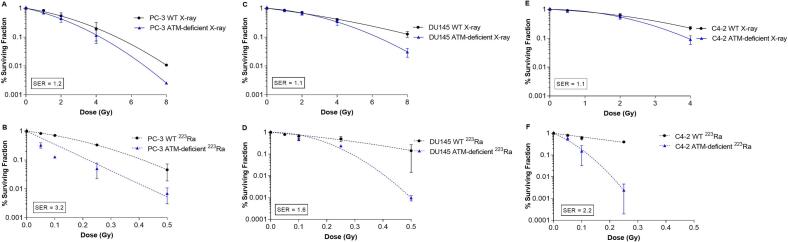
Clonogenic survival curves of PCa cells following X-rays or ^223^Ra treatment. Survival curves for PC-3 (A,B), DU145 (C,D) and C4-2 (E,F) PCa WT and ATM-deficient cell lines after exposure to X-rays (0-8 Gy) (A,C,E) or ^223^Ra (0-0.5 Gy) (B,D,F) for 24 h. Colonies were counted after 7 days of culture and survival fractions calculated. All survival curves are fitted to a linear quadratic model and sensitisation enhancement ratios (SER) were calculated from the ratios of the mean inactivation dose (MID). Points represent the mean ± respective standard error from three independent experiments.

To investigate the impact of ATM loss on radiosensitivity across different radiation modalities, clonogenic survival assays were performed using isogenic ATM-deficient and WT PCa cells (PC-3, DU145 and C4-2) (Fig. 1). For both radiation modalities, ATM deficiency led to a greater reduction in survival in comparison to WT cells, with a more pronounced effect observed following ^223^Ra exposure in comparison to X-rays (Supplementary Table S1). This differential radiosensitisation response is reflected in the calculated SERs, with ATM-deficient cells showing the greatest increase following ^223^Ra exposure (PC-3: 3.2; DU145: 1.6; C4.2: 2.2) in comparison to X-ray treatment (PC-3: 1.2; DU145: 1.1; C4.2: 1.1). Across all PCa models, ^223^Ra exhibited greater cell killing efficacy relative to X-rays, as reflected by the RBE values (Supplementary Table S1), which is consistent with previously reported data [17], [24], [25]. Notably, this effect was more pronounced in ATM-deficient cells, which exhibited ∼ 2 times higher RBE values relative to their WT counterparts.

**Fig. 2:**
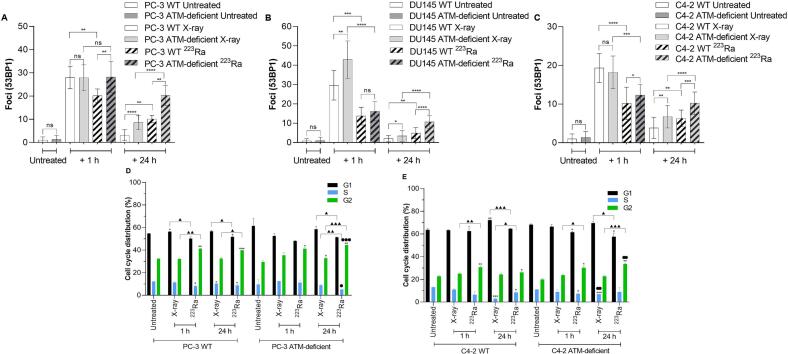
Induction of DNA damage and cell cycle distribution in PCa cells following X-rays or ^223^Ra treatment. Mean 53BP1 foci per cell was quantified in PC-3 (A), DU145 (B) and C4-2 (C) PCa WT and ATM-deficient cell lines at 1 h and 24 h following X-rays (2 Gy) or ^223^Ra (0.25 Gy). Cell cycle profiles of PC-3 (D) and C4-2 (E) cells assessed at corresponding timepoints. Bars represent the mean ± respective standard error from three independent experiments. Statistical significance was determined using an unpaired Student’s *t*-test (**** *p* < 0. 0001; *** *p* < 0. 001; ** *p* < 0.01; * *p* < 0.05; non-significant (ns)). For cell cycle distribution: respective untreated controls vs. all groups *p* < 0.05-0.001; corresponding timepoint and cell line for X-ray vs. ^223^Ra ^▲^*p* < 0.05-0.001; corresponding radiation modality and timepoint for WT vs. ATM-deficient cell line ^●^*p* < 0.05-0.001.

To explore the underlying mechanisms of enhanced radiosensitivity in ATM-deficient PCa cells, we next assessed DNA damage induction and repair using immunofluorescence detection of 53BP1 foci (Fig. 2 A-C), with ^223^Ra-treated cells analysed 1 h or 24 h following completion of the 24 h exposure period. Both radiation modalities induced a significant increase in 53BP1 foci at 1 h in comparison to untreated controls (*p* < 0.0001 for all conditions), with acute 2 Gy X-ray exposure inducing more foci than 0.25 Gy ^223^Ra delivered continuously over 24 h. This may reflect differences in radiation track structure and exposure geometry, as ^223^Ra in solution results in α-particle tracks impinging on cells from multiple directions, whereas overlapping foci following external beam irradiation may reduce the number of individually distinguishable foci. By 24 h, a large proportion of 53BP1 foci had resolved in the PCa WT cells following X-ray treatment, indicating the effective repair of DSBs. In contrast, residual foci at 24 h remained after ^223^Ra exposure, even in the presence of functional ATM. However, ATM-deficient cells retained significantly higher 53BP1 foci following ^223^Ra exposure in comparison to X-rays (ATM-deficient ^223^Ra vs. X-rays foci/cell; PC-3 20.2 ± 4.3 vs. 8.6 ± 3.1; DU145 10.7 ± 3.2 vs. 3.4 ± 2.8; C4-2 10.2 ± 2.9 vs. 6.8 ± 2.9) and their WT counterparts (WT vs. ATM-deficient after ^223^Ra exposure foci/cell; PC-3 10.1 ± 1.6 vs. 20.2 ± 4.3; DU145 4.9 ± 2.8 vs. 10.7 ± 3.2; C4-2 6.3 ± vs. 10.2 ± 2.9) (*p* < 0.001 for all conditions), indicating loss of ATM enhances the accumulation of unrepaired, complex DNA damage, underscoring the greater repair challenge posed by high LET α-particle irradiation. (Representative images in Supplementary Fig. 2).

Given the increased DNA damage observed in ATM-deficient cells, we examined whether these effects were associated with alterations in cell cycle progression (Fig. 2 D&E). Across all cell lines and time points examined, the proportion of cells in G2 phase were significantly increased after ^223^Ra treatment compared with untreated controls (*p* < 0.05 for all conditions). A direct comparison of the radiation modalities at 24 h post treatment, revealed a significant G2 arrest in PC-3 wt (*p* < 0.05), PC-3 ATM-deficient (*p* < 0.001) and C4-2 ATM-deficient cells (*p* < 0.05) accompanied by a corresponding decrease in the G1 populations. However, G2 accumulation was greatest in ATM-deficient cells following ^223^Ra exposure in comparison to X-rays (ATM-deficient ^223^Ra vs. X-rays G2 population; PC-3 44.4 ± 0.1 vs. 32.8 ± 1.1; C4-2 33.5 ± 0.6 vs. 22.8 ± 0.9) and WT counterparts (WT vs. ATM-deficient after ^223^Ra exposure G2 population; DU145 39.9 ± 1.3 vs. 44.4 ± 0.1; C4.2 26.1 ± 0.7 vs. 33.5 ± 0.6) (*p* < 0.01 for all conditions).

**Fig. 3:**
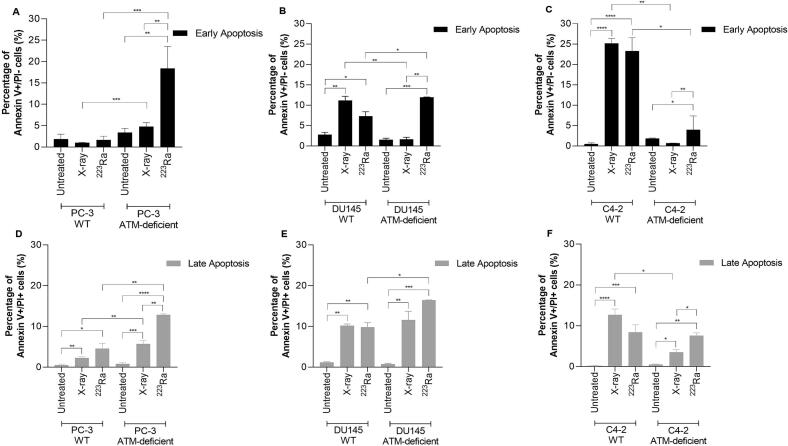
Quantification of apoptotic cells in PCa cell lines following X-rays or ^223^Ra treatment. Quantification of the percentage of early (A-C) and late (D-F) apoptosis induction in PC-3 (A,D), DU145 (B,E) and C4-2 (C,F) PCa WT and ATM-deficient cell lines 96 h after exposure to X-rays (2 Gy) or ^223^Ra (0.25 Gy). Annexin V+/PI- populations were classified as early apoptotic cells, while Annexin V+/PI+ populations represent late apoptosis or secondary necrosis. Bars represent the mean ± respective standard error from three independent experiments. Statistical significance was determined using an unpaired Student’s *t*-test (**** *p* < 0. 0001; *** *p* < 0. 001; ** *p* < 0.01; * *p* < 0.05).

Given the observed DNA damage and G2 phase arrest after irradiation, we next tested whether ATM loss influenced cell death by quantifying early and late apoptosis 96 h after exposure to X-rays or ^223^Ra (Fig. 3). For early apoptosis, PC-3 wt cells demonstrated no significant change following either radiation modality in comparison to untreated controls, whereas PC-3 ATM-deficient cells displayed a significant increase after ^223^Ra exposure (*p* < 0.01) but not after X-ray treatment. The greatest early apoptotic cell fraction was in PC-3 ATM-deficient cells treated with ^223^Ra (18.35 ± 5.1%). In DU145 and C4-2 WT cells, both radiation modalities induced significant increases in early apoptosis relative to untreated controls (*p* < 0.05 for all conditions). In DU145 ATM-deficient cells, ^223^Ra produced the greatest early apoptotic response in comparison to untreated controls (*p* < 0.001), while X-rays elicited a significantly smaller early apoptotic cell fraction in comparison to WT counterparts (1.65 ± 0.5% vs. 11.2 ± 0.9% (*p* < 0.01)). In C4-2 cells the WT cell line exhibited the highest levels of early apoptosis, with X-rays and ^223^Ra producing similar apoptotic fractions (25.15 ± 1.2% and 23.23 ± 3.3%, respectively (*p* < 0.0001)). In contrast, C4-2 ATM-deficient cells showed significantly lower levels of early apoptotic fractions after both treatments in comparison to WT cells (*p* < 0.05 for all conditions).

Assessment of late apoptosis revealed that both PC-3 and DU145 WT and ATM-deficient cells exhibited significant increases in the fraction of late apoptotic cells following both radiation modalities relative to their corresponding untreated controls (*p* < 0.05 for all conditions). The greatest effect was observed in ATM-deficient cells treated with ^223^Ra, where the fraction of late apoptotic cells increased from 4.53 ± 1.3% to 12.8 ± 0.3% in PC-3 ATM-deficient cells and from 9.8 ± 1.1% to 16.45 ± 0.1% in DU145 ATM-deficient cells relative to WT counterparts. In C4-2 WT cells, X-rays induced the strongest apoptotic response relative to ^223^Ra and ATM-deficient cells. No significant difference was observed between C4-2 WT and ATM-deficient cells following ^223^Ra exposure, with both showing comparable fractions of late apoptotic cells (8.4 ± 1.8% vs. 7.6 ± 0.7%, respectively).

**Fig. 4:**
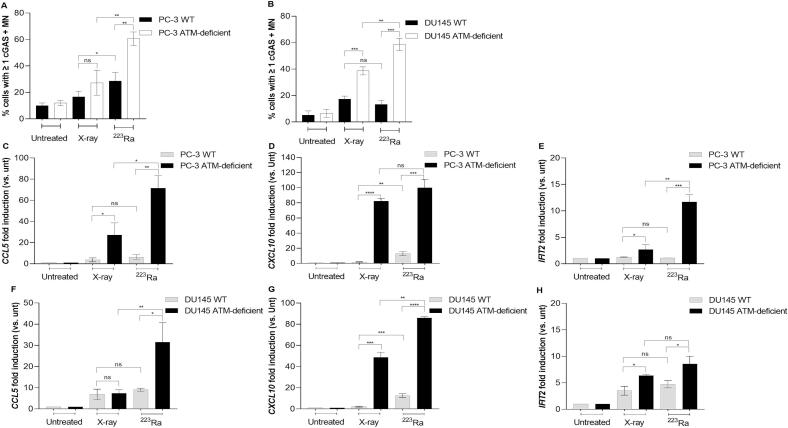
Induction of cGAS and mRNA levels of STING driven inflammatory genes in PCa cells following X-rays or ^223^Ra treatment. Percentage of PC-3 (A) and DU145 (B) PCa WT and ATM-deficient cell lines with one or more cGAS positive micronuclei determined by immunofluorescent cGAS staining at 96 h post-irradiation. qPCR analysis of *CCL5* (C,F), *CXCL10* (D,G), and *IFIT2* (E,H) mRNA expression in PC-3 (C-E) and DU145 (F-H) PCa WT and ATM-deficient cell lines at 96 h following X-rays (2 Gy) or ^223^Ra (0.25 Gy). Bars represent the mean ± respective standard error from three independent experiments. Statistical significance was determined using an unpaired Student’s *t*-test (**** *p* < 0. 0001; *** *p* < 0. 001; ** *p* < 0.01; * *p* < 0.05; non-significant (ns)).

Next, we examined whether ATM loss modulates the cGAS-STING pathway in response to different radiation modalities. cGAS is an important DNA sensor that produces cyclic 2,3-GMP–AMP (cGAMP) upon detecting cytosolic and micronuclear double-stranded DNA, thereby activating STING and downstream type I interferon signalling [26]. To assess this mechanism, we performed immunofluorescent cGAS staining to quantify the percentage of cells containing one or more cGAS-positive micronuclei following X-ray or ^223^Ra treatment, providing a measure of micronuclear DNA/cGAS accumulation and pathway activation (Fig. 4 A&B). DU145 ATM-deficient cells exhibited a significantly higher proportion of cGAS-positive micronuclei compared to WT cells after both X-ray and ^223^Ra exposure (*p* < 0. 001), whereas in the PC-3 isogenic cell line, this difference was only significant after ^223^Ra treatment (*p* < 0.01). Across both cell lines, cGAS co-localisation with micronuclei was more pronounced following ^223^Ra in ATM-deficient cells (*p* < 0.01 for all conditions). Taken together, these findings support a model in which clustered α-particle-induced DNA lesions generate persistent micronuclei that activate cGAS, particularly under conditions of impaired DSB repair caused by ATM loss.

Previous studies in other cancer models have reported that ATM loss promotes the upregulation of immune-related genes after radiation, suggesting that ATM-deficient cells activate cGAS-STING signalling after irradiation [27]. Consistent with this, ATM-deficient PC-3 and DU145 cells exhibited markedly greater upregulation of the STING driven inflammatory genes *CCL5*, *CXCL10* and *IFIT2* following X-rays (Fig. 4 C-H). This increase was statistically significant across all transcripts (*p* < 0.05 for all conditions), except for *CCL5* in DU145 cells, where expression levels were comparable between WT and ATM-deficient cells. However, ^223^Ra exposure elicited a stronger induction of these genes, particularly in ATM-deficient cells, with both PC-3 and DU145 ATM-deficient cells demonstrating significantly higher fold induction of all three STING driven chemokine genes, relative to their WT counterparts (*p* < 0.05 for all conditions). (Representative images in Supplementary Fig. 3).

## Discussion

The treatment landscape for mCRPC is rapidly evolving, with the development and application of personalised strategies gaining significant clinical attention. Genomic alterations of ATM, including frameshift and missense mutations as well as complete gene loss, are observed in approximately 5% of advanced prostate cancer tumours, supporting the clinical relevance of ATM deficiency as a molecularly defined subgroup. Consistent with this, enhanced clinical response to ^223^Ra has been observed in metastatic patients with HRR-mediated DNA repair gene deficiencies, including ATM loss, indicate that DDR defects increase radiosensitivity to the α-emitter ^223^Ra [15], [16]. To further explore this rationale, the present study characterised the mechanistic basis by which ATM loss in PCa *in vitro* models modulated cellular response to X-rays and ^223^Ra. Our findings demonstrate that ATM-deficiency enhances both the radiobiological and cGAS-STING-driven immune activation to ^223^Ra.

ATM-deficiency markedly increased cellular radiosensitivity across both radiation modalities, with the greatest response observed after ^223^Ra exposure. This data is consistent with previous studies which have reported pharmacological inhibition of ATM to potentiate the efficacy of ^223^Ra in PCa models [17], [28]. These findings are also in agreement with the crucial role ATM plays in orchestrating DNA DSB repair, checkpoint signalling and maintaining genomic integrity [29], [30]. However, the magnitude of radiosensitisation observed following X-irradiation was modest relative to some previously reported models with pharmacological ATM blockade [31]. Although ATM protein expression was undetectable by Western blotting, residual ATM signalling activity cannot be excluded and may have contributed to the reduced SER values observed in these models. Further studies using pharmacological ATM inhibition may help clarify this possibility.

The reduced survival associated with ^223^Ra may be attributed to complex, spatially clustered DNA DSBs, which are characteristically induced by α-emitters and are increasingly difficult for DNA repair machinery to resolve in comparison to DNA lesions induced by X-rays, particularly in the absence of ATM. This observation is consistent with our data, whereby ^223^Ra induced DNA DSBs that exhibited reduced repair efficiency in ATM-deficient PCa cells, as supported by the persistence of significantly greater 53BP1 foci observed at 24 h.

Once activated by radiation-induced DNA damage, ATM phosphorylates (Thr68) and activates Chk2, activating a signalling cascade which ultimately leads to cell cycle arrest through inhibition of cyclin/CDK complexes, thereby enabling the repair of DNA DSBs [32], [33]. However, in the absence of ATM, checkpoint regulation is compromised, permitting cells with unresolved DNA damage to progress through the cell cycle which ultimately results in cell death through mitotic catastrophe or apoptosis [34]. Our findings support previously published data that high and low LET radiation causes differential changes in cell cycle distribution [31]. Examination of all cell lines determined that X-irradiation only elicited a significant accumulation of PC-3 ATM-deficient cells in G2/M, reflecting less complex DNA damage that is typical of low LET irradiation. In contrast, following ^223^Ra, all cell lines exhibited prolonged checkpoint activation and significant G2/M checkpoint arrest, with the most pronounced G2/M arrest observed in ATM-deficient cells, consistent with the complex DNA damage and reduced repair capacity associated with ^223^Ra exposure. Consistent with this mechanism, ^223^Ra yielded the greatest apoptotic response in PC-3 and DU145 ATM-deficient cells, which is in agreement with previous studies reporting increased apoptosis following ATM inhibition and ^223^Ra treatment in PCa models [17]. In contrast, the reduced apoptotic response of C4-2 ATM-deficient cells to both radiation modalities may be attributed to retained androgen receptor signalling capacity and AR activity has been reported to attenuate apoptotic engagement, while shifting cell death towards mitotic catastrophe or senescence-like outcomes. This may explain the divergent phenotype relative to PC-3 and DU145 and warrants further mechanistic investigation [35], [36], [37].

PCa is frequently considered “immunologically” cold, primarily due to its low mutational burden and immunosuppressive tumour microenvironment (TME). However, emerging evidence has demonstrated that both high LET irradiation or loss/pharmacological inhibition of DDR genes enhances antitumour activity by activating the cGAS-STING signalling pathway in PCa [38], [39], [40]. In this study we identified an increase in cGAS accumulation at micronuclei and concomitant transcriptional activation of STING driven inflammatory genes in ATM-deficient cells after both radiation modalities, with the most prominent response observed after ^223^Ra exposure. In agreement with our data, studies in multiple tumour types have reported ATM inhibition to significantly enhance radiation-induced cGAS-STING activation, cytokine production and TME modulation [39], [41], [42]. However, to the best of our knowledge, this is the first preclinical study to demonstrate that ATM loss amplifies α-particle-induced innate immune signalling, where these data indicate that ATM loss not only increases radiobiological sensitivity to α-particle-induced DSBs, but also enhances downstream cytosolic DNA sensing and type I interferon activation, suggesting potential synergy between ^223^Ra and immunotherapy approaches in ATM-deficient disease.

In conclusion, we have demonstrated that loss of ATM markedly increased cellular radiosensitivity to α-particle irradiations with ^223^Ra through impaired DNA DSB repair, sustained G2 arrest and increased apoptosis. Furthermore, upregulation of the cGAS-STING-mediated immune response in ATM-deficient cells following ^223^Ra treatment indicates the potential of DDR loss to enhance tumour immunogenicity. Together, these findings offer mechanistic insight into the enhanced RBE of ^223^Ra in ATM-deficient PCa in vitro models and provide a preclinical basis for further exploration of ATM deficiency in the context of α-radiopharmaceutical-based therapies and potential immunotherapy combination strategies.

## Funding Sources

VLD is supported by a Prostate Cancer UK Career Acceleration Fellowship [Grant Number TLD-PF21-001]. T.C.W. and V.L.D. are also supported by the LFT Charitable Trust. KIS is supported by Cancer Research UK (A29392). Additional support was provided by the Movember/Prostate Cancer UK Centre of Excellence [CEO13_2-004] and the Research and Development Division of the Public Health Agency of Northern Ireland [COM/4965/14].

## CRediT authorship contribution statement

**Victoria L. Dunne:** Writing – review & editing, Writing – original draft, Visualization, Validation, Resources, Project administration, Methodology, Investigation, Funding acquisition, Formal analysis, Data curation, Conceptualization. **Timothy C. Wright:** Writing – review & editing, Methodology, Investigation. **Aislinn Toner:** Writing – review & editing, Methodology, Investigation. **Melissa LaBonte Wilson:** Writing – review & editing, Methodology, Formal analysis. **Kienan I. Savage:** Writing – review & editing, Supervision, Methodology, Formal analysis, Conceptualization. **Joe M. O’Sullivan:** Writing – review & editing, Supervision, Project administration, Methodology, Funding acquisition, Conceptualization. **Kevin M. Prise:** Writing – review & editing, Supervision, Resources, Project administration, Funding acquisition, Conceptualization.

## Declaration of competing interest

The authors declare that they have no known competing financial interests or personal relationships that could have appeared to influence the work reported in this paper.
