# Supplementary material for: Impact of ataxia-telangiectasia mutated (ATM) loss on radiobiological and immune response to radium-223 in prostate cancer in vitro models
Source: Clin Transl Radiat Oncol. 2026 May 30;59:101206. doi: 10.1016/j.ctro.2026.101206 (PMC13254887; doi:10.1016/j.ctro.2026.101206)
Supplement: Supplementary Data 1 — Supplementary Methods and Data. [file mmc1.docx]

**Supplementary Methods**

*Cell Lines and Cell Culture*

PC-3 and DU145 CRISPR-Cas9-mediated ATM-deficient isogenic human PCa cell lines were kindly provided by Dr. Susan P. Lees-Miller (University of Calgary, Canada) and Dr Kent W. Mouw (Dana-Farber Cancer Institute, Harvard Medical School, USA) [1,2]. C4-2 ATM-deficient cells were generated at Queen’s University Belfast using CRISPR-Cas9 technology [3] (see Supplementary Fig 1). PC-3 cells were cultured in F-12K nutrient mixture (1×) Kaighn’s modification (ThermoFisher Scientific, Waltham, MA, USA) and DU145 and C4-2 cells were cultured in RPMI-1640 (Gibco, Thermo Fisher Scientific, Waltham, MA, USA). All cells were supplemented with 10% fetal bovine serum (FBS) (Sigma-Aldrich, St. Louis, MO, USA) and 1% pencillin-streptomycin (Thermo Fisher Scientific). All cell lines were routinely tested for mycoplasma.

*Irradiation Setups*

X-ray irradiation (0–8 Gy) was performed using an X-Rad 225 system (2 mm copper filter; Precision X-RAY Inc., North Branford, CT, USA) at a dose rate of 0.57 Gy/min.

For ²²³Ra exposures, Xofigo® (Bayer, Leverkusen, Germany), activities ranging from 0.1 to 0.5 MBq/mL was kindly provided by the Northern Ireland Cancer Centre. The volume of ²²³Ra solution added to each well was calculated via microdosimetry, taking into account vial activity, exposure time and target absorbed dose, as previously described [4]. Cells in 6-well plates were exposed to 0–0.5 Gy by adding up to 50 μL of Xofigo to 2 mL of culture medium with an exposure time of 24 h. To maintain a consistent total volume across all conditions, ²²³Ra solutions were pre-diluted in saline (0.1 M NaCl, 0.02 M Na₃C₆H₅O₇, 5 mM HCl, pH 7.0; Sigma-Aldrich, Glasgow, UK), matching the volume of the Xofigo solution added to the highest treatment. The average alpha particle emission energy from the ²²³Ra decay cascade is 6.67 MeV, with an entrance LET of 72 keV/μm. Control samples received the same saline solution without ²²³Ra. After exposure, cells were washed and incubated in fresh medium. Although the absorbed doses delivered in monolayer culture do not directly replicate the heterogeneous micro-dosimetry of bone metastases i*n vivo*, the dose range used in this study reflects established *in vitro* models of α-emitter radiobiology and enables comparison of intrinsic repair and survival responses between genotypes.

*Immunoblotting*

Cells were harvested and lysed 24 h following seeding, according to established protocols [5]. Protein lysates (40 µg per sample) were loaded onto NuPAGE 8% Bis-Tris gels (Invitrogen) and transferred onto PVDF membranes using the iBlot2 system (Invitrogen). Membranes were blocked and incubated overnight at 4 °C with primary antibodies against ATM (#23921, Santa Cruz Biotechnology, Dallas, TX, USA). β-actin (#4967, Cell Signaling Technology; 1:5000). After washing in PBS-T, membranes were incubated for 1 h at room temperature with anti-anti-mouse HRP-conjugated secondary antibodies (1:2000). Protein bands were visualized using enhanced chemiluminescence (ECL) and imaged with a G:BOX system (Syngene, Cambridge, UK).

**Supplementary Material**

Impact of ataxia-telangiectasia mutated (ATM) loss on radiobiological and immune response to radium-223 in prostate cancer *in vitro* models.

Victoria L. Dunne, Timothy C. Wright, Aislinn Toner, Melissa LaBonte Wilson, Joe M. O’Sullivan and Kevin M. Prise.

**Supplementary Table 1.** **Linear quadratic parameters for PCa cell lines.** PC-3, DU145 and C4-2 PCa WT and ATM-deficient cell lines after exposure to different radiation modalities.

| **Parameters** | | **PC-3**  **WT** | **PC-3**  **ATM-deficient** | **DU145 WT** | **DU145 ATM-deficient** | **C4-2 WT** | **C4-2 ATM-deficient** |
| --- | --- | --- | --- | --- | --- | --- | --- |
| X-ray | α (Gy^-1^) | 0.22 ±  0.06 | 0.32 ±  0.05 | 0.12 ±  0.03 | 0.12 ±  0.04 | 0.12 ±  0.06 | 0.01 ±  0.1 |
|  | β (Gy^-2^) | 0.04 ±  0.01 | 0.05 ±  0.01 | 0.01 ± 0.003 | 0.04 ±  0.01 | 0.01 ±  0.02 | 0.1 ±  0.03 |
| ^223^Ra | α (Gy^-1^) | 2.8 ±  0.8 | 10.5 ±  2.07 | 2.6 ±  1.5 | ~ 0 | 3.9 ±  0.9 | 12.5 ±  4.4 |
|  | β (Gy^-2^) | 6.8 ±  1.9 | ~ 0 | 2.5 ±  3.2 | 27.7 ±  2.4 | ~ 0 | 46.4 ±  19.2 |
| RBE  (X-ray/^223^Ra) |  | 13.22 | 34.06 | 15.54 | 21.96 | 15.85 | 31.71 |


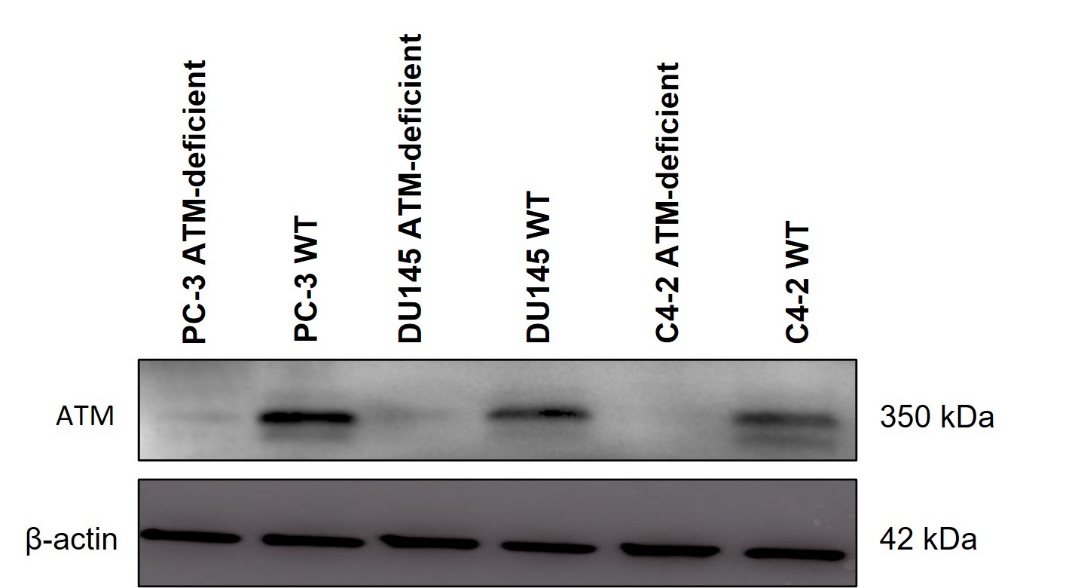


**Fig. S1.** Western blot validation confirming loss of ATM protein expression in PC-3, DU145 and C4-2 PCa WT and ATM-deficient cell lines.


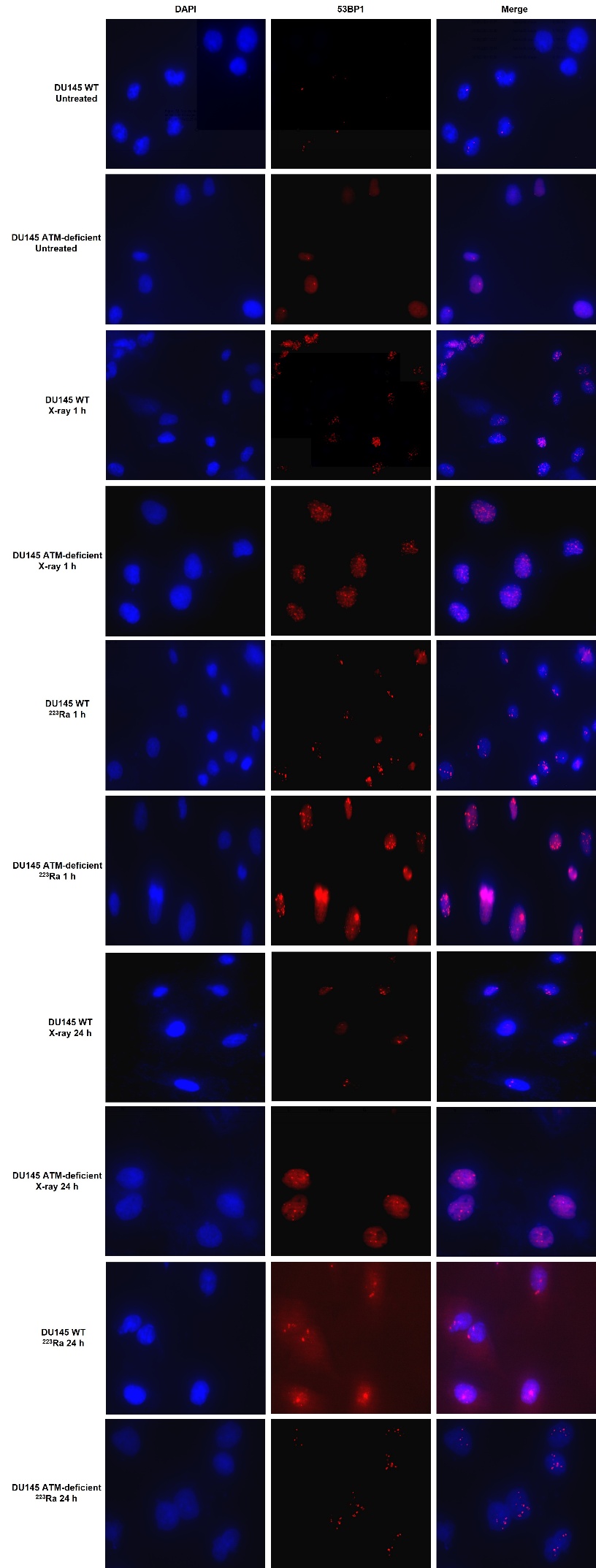


**Fig. S2.** Representative images of mean 53BP1 foci per cell for DU145 PCa WT and ATM-deficient cells following no treatment, X-rays (2 Gy) or ^223^Ra (0.25 Gy) at 1 h and 24 h timepoints.


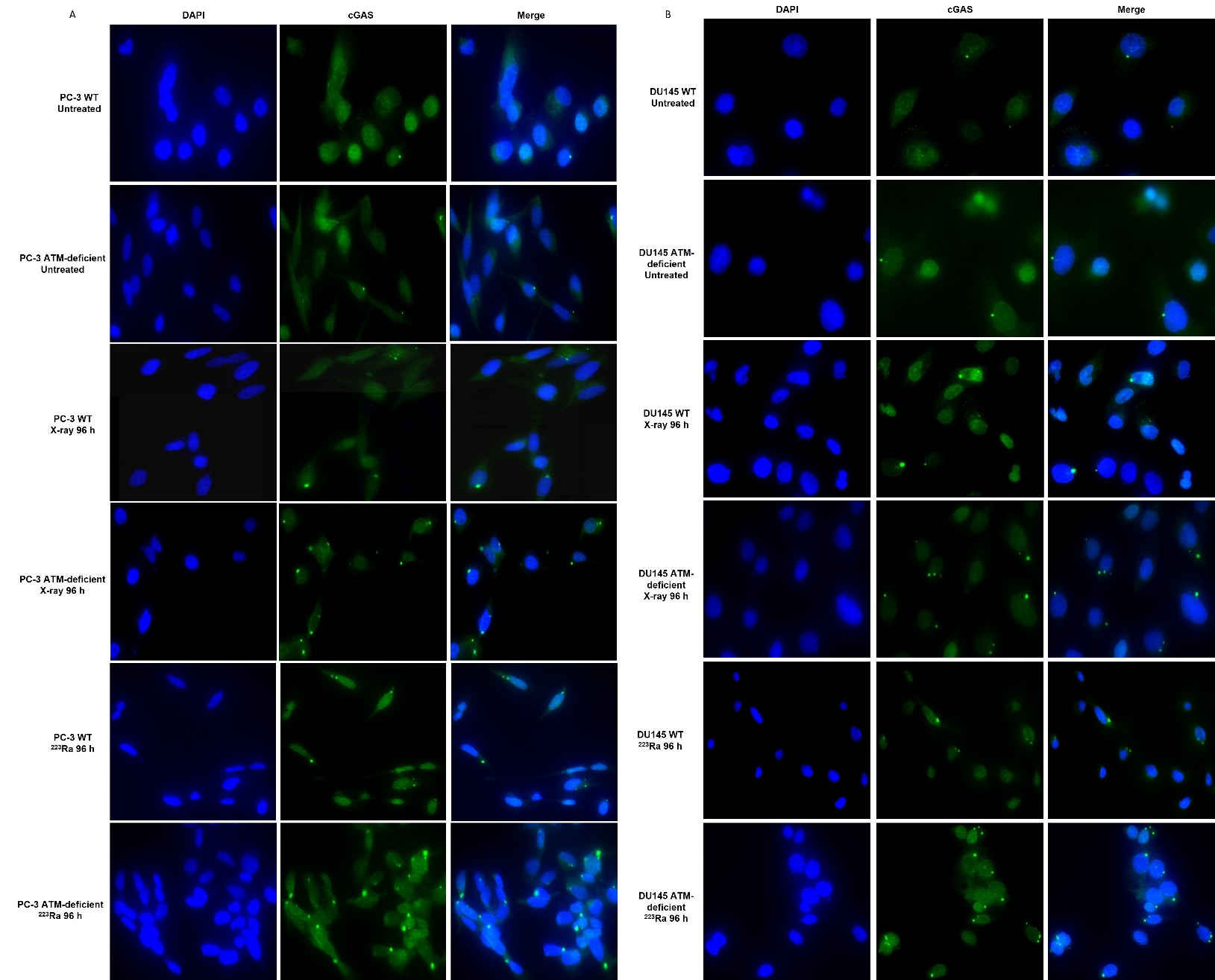


**Fig. S3.** Representative images of PC-3 (**A**) and DU145 (**B**) PCa WT and ATM-deficient cell lines with cGAS staining following no treatment, X-rays (2 Gy) or ^223^Ra (0.25 Gy) at 96 h.

**References**

1. Rafiei S, Fitzpatrick K, Liu D, Cai M-Y, Elmarakeby HA, Park J, et al. ATM Loss Confers Greater Sensitivity to ATR Inhibition Than PARP Inhibition in Prostate Cancer. Cancer Research 2020;80:2094-2100. https://doi.org/[10.1158/0008-5472.CAN-19-3126](https://doi.org/10.1158/0008-5472.can-19-3126)
2. Jette NR, Radhamani S, Ye R, Yu Y, Arthur G, Goutam S, et al. ATM-deficient lung, prostate and pancreatic cancer cells are acutely sensitive to the combination of olaparib and the ATR inhibitor AZD6738. Genome Instability & Disease 2020;1:197-205. https://doi.org/10.1007/s42764-020-00011-0
3. Liberal FDCG, McMahon SJ. Characterization of Intrinsic Radiation Sensitivity in a Diverse Panel of Normal, Cancerous and CRISPR-Modified Cell Lines. International Journal of Molecular Sciences 2023;24:7861. https://doi.org/[10.3390/ijms24097861](https://doi.org/10.3390/ijms24097861)
4. Guerra Liberal FDC, Moreira H, Redmond KM, O’Sullivan JM, Alsherhi AHD, Wright TC, et al. Differential responses to 223Ra and Alpha-particles exposure in prostate cancer driven by mitotic catastrophe. Frontiers in Oncology 2022;12. https://doi.org/[10.3389/fonc.2022.877302](https://doi.org/10.3389/fonc.2022.877302)
5. Schumann S, Eberlein U, Muhtadi R, Lassmann M, Scherthan H. DNA damage in leukocytes after internal ex-vivo irradiation of blood with the α-emitter Ra-223. Scientific Reports 2018;8. https://doi.org/10.1038/s41598-018-20364-7
